# Recent Advances on Composition-Microstructure-Properties Relationships of Precipitation Hardening Stainless Steel

**DOI:** 10.3390/ma15238443

**Published:** 2022-11-27

**Authors:** Puchang Cui, Geshu Xing, Zhisheng Nong, Liang Chen, Zhonghong Lai, Yong Liu, Jingchuan Zhu

**Affiliations:** 1 School of Materials Science and Engineering, Harbin Institute of Technology, Harbin 150001, China; 2 School of Materials Science and Engineering, Shenyang Aerospace University, Shenyang 110136, China; 3 Aero Engine Corporation of China Gas Turbine Co., Ltd., Shenyang 110623, China; 4 Center for Analysis, Measurement and Computing, Harbin Institute of Technology, Harbin 150001, China; 5 National Key Laboratory for Precision Hot Processing of Metals, Harbin Institute of Technology, Harbin 150001, China

**Keywords:** precipitation hardening stainless steel, composition-microstructure-properties relationships, multi-scale simulation, machine learning, alloy design and characterization

## Abstract

Precipitation hardening stainless steels have attracted extensive interest due to their distinguished mechanical properties. However, it is necessary to further uncover the internal quantitative relationship from the traditional standpoint based on the statistical perspective. In this review, we summarize the latest research progress on the relationships among the composition, microstructure, and properties of precipitation hardened stainless steels. First, the influence of general chemical composition and its fluctuation on the microstructure and properties of PHSS are elaborated. Then, the microstructure and properties under a typical heat treatment regime are discussed, including the precipitation of B2-NiAl particles, Cu-rich clusters, Ni_3_Ti precipitates, and other co-existing precipitates in PHSS and the hierarchical microstructural features are presented. Next, the microstructure and properties after the selective laser melting fabricating process which act as an emerging technology compared to conventional manufacturing techniques are also enlightened. Thereafter, the development of multi-scale simulation and machine learning (ML) in material design is illustrated with typical examples and the great concerns in PHSS research are presented, with a focus on the precipitation techniques, effect of composition, and microstructure. Finally, promising directions for future precipitation hardening stainless steel development combined with multi-scale simulation and ML methods are prospected, offering extensive insight into the innovation of novel precipitation hardening stainless steels.

## 1. Introduction

Precipitation hardening stainless steel (PHSS) has a long history and is widely employed in aerospace industries, marine environment applications, and nuclear reactor fields due to its ultra-high strength, satisfactory ductility, and excellent anti-corrosion properties [1,2,3,4,5]. Mechanical properties, such as strength, ductility, toughness, and corrosion properties, including self-corrosion potential and self-corrosion current density, are several important descriptors of metallic structural materials. Ensuring excellent mechanical properties and the anti-corrosion properties of the ultra-high stainless steel to the environment is a key factor for the long-term life of structural part materials in the actual service environments [6,7]. Ultimately, PHSS can achieve all its advantages. However, with the increasing requirements of PHSS in the harsher service environment, there is an urgent need for alloy design to meet the iterations of advanced structural stainless steels.

The microstructure and distinguished properties of ultra-high strength stainless steels have been characterized through experimental techniques in the past decades [8,9,10]. The composition of steel was proportioned, then the microstructure and properties were enhanced and the relationships among them were established through traditional experimental processing; however, the rapid composition design and property optimization cannot be achieved. Great attempts have been focused on the establishment of composition-microstructure-properties relationships through conventional trial and error experimental investigations, which are time- and resource-consuming. It has been proven that the precipitated phase and reversed austenite play a key role in mechanical and corrosion properties. The sequential formation of segregated phases in PH17-4 PHSS was observed at two different aging temperatures [11]. The Cu-rich precipitates in the martensitic matrix mainly determine the mechanical properties rather than the reverted austenite from the mechanical testing results [12,13]. A. Barroux et al. have investigated that the pitting corrosion behavior of the PH17-4 steel fabricated by laser beam melting methods is better than the traditional PH17-4 steel [14]. Ronald Schnitzer et al. have demonstrated that the reverted austenite in PH13-8Mo steel aged at 575 °C shows two types forms, and the precipitation of B2-NiAl precipitates and the formation of reversed austenite occur simultaneously. Meanwhile, the enhancement of the strengthening capability of B2-NiAl precipitates is related to the growth of B2-NiAl precipitates and the formation of B2-NiAl precipitates alters the strain rate sensitivity [15,16,17]. Furthermore, it is necessary to further rapidly model the relationship among alloy composition, process/microstructure, and properties. Multi-scale simulation and ML techniques of materials play a vital role in the development of novel materials. The capability of ML largely originates from its statistical analysis of big data, which comes from experiments, first principles calculations, and molecular dynamics (MD) simulations [18,19,20]. In addition, ML can not only carry out alloy design, but also realize many important functions, such as image recognition based on pattern digitization techniques [21,22,23].

Various martensite variant microstructures with significant anisotropy features were demonstrated based on first principles calculation and phase-field simulation methods, which is in acceptable agreement with experimental results [24,25]. The behavior and mechanisms of hydrogen embrittlement were revealed in high Co-Ni secondary hardening steel, which exhibited the improving accuracy of multi-scale simulation approaches [26]. Sayyed Ali Razavi et al. have carried out the predication and optimization of aging strengthening parameters using an artificial neural network (ANN) combined with genetic algorithm in PH17-4 steel and the consistency between the hardness experiment and the prediction results shows that the proposed model is significantly effective [27]. Therefore, it is imperative to design PHSS with the assistance of ML approaches.

In this review, we concentrate on recent progress in the advancement of PHSS, including the ultra-high strength steel not limited to the precipitation hardening steel (PHS) and PHSS, in regard to the modeling of composition-microstructure-properties relationships, especially in the hot working process, as well as the effects of compositions on the microstructure and properties of PHSS steels. Although the PHSS has a relatively long history, we would like to present how to establish a quantitative model of composition-structure-property to quickly discover novel high-performance PHSS. We start by summarizing the composition development and property features of PHSS, the microstructures and properties under representative heat treatments, and then discussing the microstructures and properties of the PHSS prepared by the selective laser melting (SLM) method. Finally, the strategies of alloy design and tailored property optimization of PHSS are prospected. This work is devoted to providing useful insight into the design strategies that are used to develop novel high-performance PHSS, specifically for engineering applications.

## 2. Effects of Chemical Composition and Its Fluctuation on Microstructure and Properties

**Figure 1:**
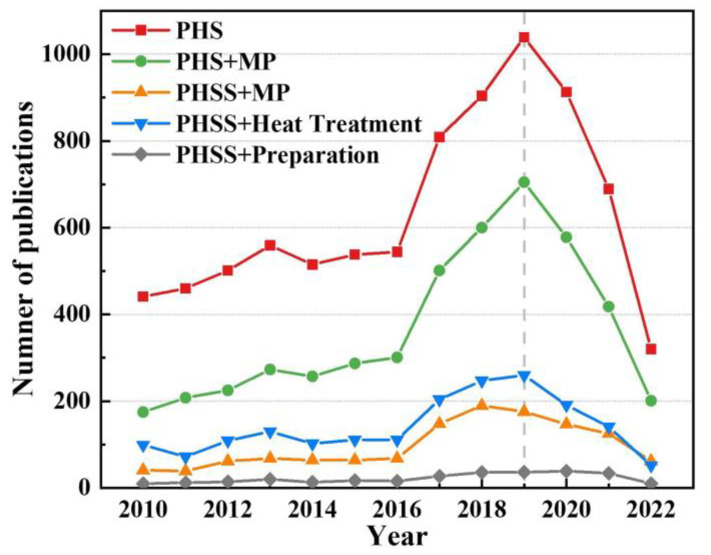
Number of precipitation hardening stainless steel publications per year with microstructure-properties, heat treatment, and preparation since 2010 (data from Web of Science database until 16 September 2022).

Many investigations on PHSS have been carried out, and most of them focus on mechanical and corrosion behavior [12,16,28,29,30,31,32]. Tian et al. [28] have developed a novel stainless steel with distinguished strength and toughness balance properties and acceptable anti-corrosion properties. The excellent mechanical properties originate from the precipitation effect, and the corrosion property is achieved by regulating the Co and Cr contents. The aging heat treatment can clearly enhance the anti-corrosion properties of the sintered PH17-4 PHSS in dilute sulfuric acid environment and the aging temperature of 480 °C can obtain the best anti-corrosion properties [29]. The results help us in better understanding the composition-microstructure-property relationship of various steels. Concerning research directions in PHSS, we present a brief statistical overview of the annual publications on precipitation-hardening stainless steel retrieved with keywords of “precipitation hardening steel (named as PHS)”, “precipitation hardening steel and microstructure property (designated as PHS + MP)”, “precipitation hardening stainless steel and microstructure property (named as PHSS + MP)”, “precipitation hardening stainless steel and heat treatment (named as PHSS + Heat Treatment)”, “precipitation hardening stainless steel and preparation (named as PHSS + Preparation)”, respectively. A distribution map of the number of annual publications on precipitation hardening steel since 2010 is illustrated in Figure 1 (data from Web of Science until 16 September 2022). It can be observed that the number of research papers on PHS has risen rapidly before 2019, which indicates that the research community is more concerned about the microstructure and mechanical properties of developed steels, and at the same time, optimizing the properties was achieved by heat treatment technologies. The number of papers on new fabrication methods of PHSS steel is small, indicating that the research in this field is in the initial stage. Additionally, it is worth noting that the number of publications on PHSS has been decreasing in the past 3 years, indicating that the potential of existing PHSS steel has reached its limit. It is necessary to develop and research new PHSS steels, especially new PHSS steels with ultra-high mechanical properties.

The first-generation precipitation hardening martensitic stainless steel was designed in the 1840s [33]. PHSS has been widely used in aerospace, marine equipment, and other fields by virtue of its extraordinary strength, toughness, and corrosive atmosphere resistance. Subsequently, PH17-4 steel was developed by Armco in 1948, which is treated by an uncomplicated heat treatment regime and possesses acceptable weldability, endowing it largely applicated in aircraft landing frames, manufacturing fasteners, and engine parts. Unfortunately, the limited cold working deformability hinders the development of PH17-4 steel. Furthermore, PH15-5 stainless steel was incubated by deliberately reducing the content of Cr element and increasing the content of Ni element in PH17-4 steel, which is currently employed as load-bearing components in aerospace and other industrial fields. On this basis, PH13-8Mo stainless steel with enhanced strength and anti-corrosion properties was developed in 1968 by further regulating the concentration of Cr and Ni elements. Ferrium S53 steel has been successfully developed based on Materials Genome Program in recent years as shown in Table 1 [34,35,36,37,38,39,40,41,42,43,44,45,46], which has been successfully applied to American A-10 fighter planes and T-38 aircraft.

**Figure 2:**
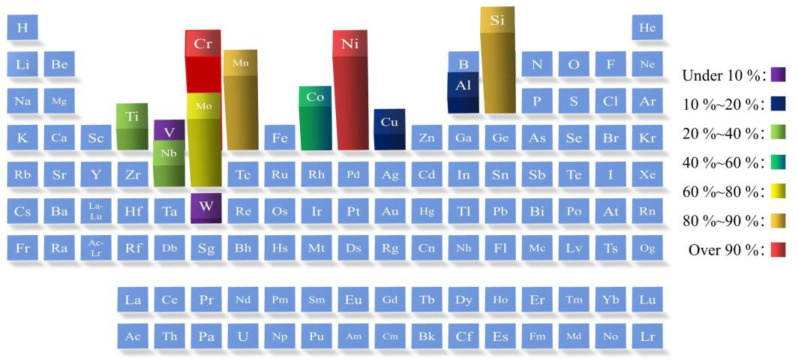
Common alloying elements with their frequency of occurrence in typical high-strength stainless steel [50,51,52,53,54,55,56,57,58].

PHSS is mainly strengthened by the precipitation of various nanoprecipitates during the aging treatment [47,48], which can significantly improve mechanical properties. Different types of nanoprecipitates tend to form via the addition of different alloying elements. At the same time, the species and density number of precipitates have significant impacts on the mechanical properties of PHSS. Moreover, it has been illustrated that the formation of oxides significantly impacts the corrosion behavior of PHSS, which is determined by the chemical composition rather than the metallurgical conditions [49]. At present, the statistical frequency of alloying elements in general ultra-high strength stainless steels is summarized in Figure 2 [50,51,52,53,54,55,56,57,58]. Meanwhile, the corresponding alloying element compositions of representative PHSS are tabulated in Table 2 [35,39,41,44,45,46,59,60,61,62,63,64,65]. Excessive efforts have been made to endeavor the investigation of effects of alloying elements on microstructures and properties over the years.

The alloying elements in ultra-high strength stainless steel are mostly concentrated in transition metallic elements, and the corresponding frequency of alloying elements is displayed in Figure 2. It can be observed that the occurrence frequency of Cr and Ni elements is up to more than 90%; namely, almost every common high-strength stainless steel contains Cr and Ni to satisfy the requirements of high strength and anti-corrosion properties. In addition, the occurrence frequency of Mo, Mn, Si, and other alloying elements is more than 60 %, which shows that these elements can have a certain positive effect on the strength of stainless steel. The general alloying strengthening elements, such as Ti, Nb, Al, Cu, etc., also occur frequently in high-strength stainless steel. Furthermore, V, W, and other alloying elements have been introduced into ultra-high strength stainless steel to improve its performance in recent years.

The corrosion properties of PHSS are inevitably associated with the chemical composition of the passive film on its surface. It is commonly deemed that the Cr concentration should be more than the threshold concentration to ensure anti-corrosion properties in typical PHSS according to the n/8 laws. Cr is one of the decisive alloying elements to enhance the promising anti-corrosion properties of PHSS [66,67,68]. However, the precipitation of intermetallic compounds will occur with the increment of Cr, which is conducive to the embrittlement of steel [69]. The proper coordination of Cr and Ni warrants the formation of a complete martensite microstructure after the quenching treatment. The addition of the Cr element promotes the precipitation of the Laves phase and other phases during the long-term aging process [70,71], which benefits the precipitation strengthening effect. Generally, the interactions of Ni and Fe tend to form ultra-low carbon content Fe-Ni martensitic microstructure in PHSS. The addition of Ni element will produce the important strengthening phase, such as the NiAl and Ni_3_Ti phases; Ni acts as a stabilizer of the austenitizing element, which promotes alloys with enhanced properties [72,73,74].

A moderate concentration of Mo is favorable for the formation of passive film on the surface of PHSS, thus boosting the pitting resistance of the steel in the Cl-containing solution. Mo element improves hardenability in PHSS, possessing the capability of resisting corrosion for stainless steel [75,76,77]. Nanosized carbide M_2_C is precipitated from the martensitic matrix in the secondary hardening steel with a balance of high strength and toughness [78,79]. However, the excessive introduction of Mo will precipitate the Mo-rich phase in steel [80].

Generally, Ti is regarded as a microalloying element and the content of Ti should be controlled within 1 wt% in PHSS. Meanwhile, as a strong carbide-forming element, Ti plays a vital role in the property regulation of steel. Therefore, it is necessary to study the effect of Ti addition on the mechanical properties of PHSS. The microstructure of Ti-containing PHSS is intricate and inclined to form intermetallic compounds. Strengthening and embrittlement behavior were observed and explained through the precipitation and evolution of precipitates [81,82]. 

A balanced Cu content in 304 stainless steel improves both the mechanical properties and anti-corrosion properties. The Cu addition in PHSS is inclined to form Cu-rich clusters distributed in the martensitic matrix uniformly. Timothy G. Lach [17] have proposed that Cu has a strong immiscibility and a strong energy interface with Fe, which has a profound impact on precipitation behavior. As a representative strengthening phase, the content of Cu in PH17-4 and PH15-5 also remains between 3% and 5% [50,83,84,85,86,87]. The explosive nucleation and growth of the Cu-rich phase significantly improve the strength of stainless steel at the initial stage of aging [88,89]. Ronald Schnitzer et al. investigated that the addition of Cu was observed to drive the precipitation of two types of precipitates. The precipitation mechanism is to reduce the activation energy and increase the nucleation site [90]. Moreover, Dieter Isheim [47,91,92] found that the Mn and Ni can be separated at the interface between the Cu-rich phase and the matrix, which is characterized by atomic probe tomography (APT) techniques. Furthermore, Kookhyun Jeong [93] and Qian Wang [94] obtained that Si and Nb elements in stainless steel can play the role of solution strengthening and grain refining strengthening, respectively. The Nb element will refine the martensitic microstructure, hinder the rapid propagation of cracks, and further enhance the stress cracking resistance of steels.

The fundamental microstructure of PHSS consists of lath-like martensitic matrix, a proper amount of austenite phase, and nano-precipitates with specific orientation relationships with the matrix, which makes the PHSS exhibit a certain balance of strength and ductility [95]. Meanwhile, the fine nanosized intermetallic phase with uniform distributions on the matrix plays a crucial role in strengthening precipitates. The mechanical properties of PHSS are closely associated with the size, quantity, and distribution of its constituent phases. In addition, the fluctuation of alloying elements directly affects the microstructure and properties of the steels. The alloying elements in PHSS form segregation zones and specific constituent phases through diffusion and redistribution after aging treatment. The formation of reverted austenite is inextricable with the diffusion of the alloying elements in PHSS under aging treatment. Advanced analysis and measurement techniques were performed to characterize the PHSS and substantive results.

**Figure 3:**
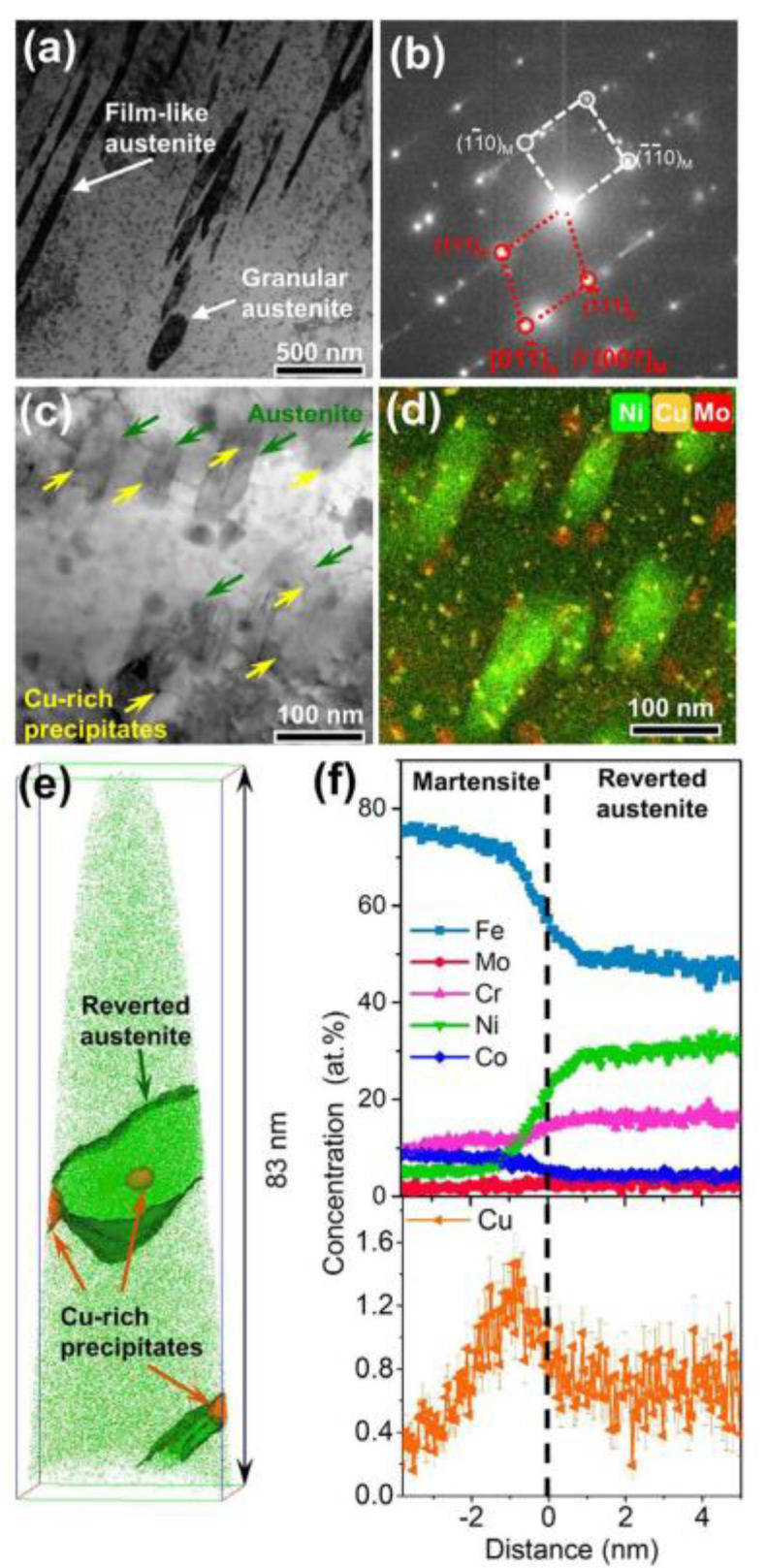
Microstructural characterization results of reverted austenite before deformation [96]: (**a**) The TEM image of the Cu-containing steel in the 60 h aging treatment, (**b**) the SAED pattern corresponding to the orientation relationship between the reverted austenite and matrix in (**a**), (**c**,**d**) are the brightfield TEM micrograph and corresponding TEM/EDS mappings of Ni, Cu, and Mo, respectively, and (**e**,**f**) are the microstructure and compositions of Cu-rich precipitates and reverted austenite employed by APT. (Reprinted with permission from Ref. [96]. Copyright 2022 Elsevier).

It has been revealed that the Cu precipitates are preferentially located at the martensite/retained austenite interfaces or martensite lath boundaries, and then the alloying elements are diffused toward the interface of the Cu precipitates. Finally, the reverted austenite is formed. At the same time, it is expounded from two aspects that the composition fluctuation of the Cu element and the solute distribution on nano-scale promote the austenite reversion transformation of martensitic stainless steel. On the one hand, Cu is regarded as an austenite stabilizer, which increases the chemical driving force for the austenite transformation in aging treatment. On the other hand, Cu-rich nanoprecipitates are served as heterogeneous nucleation sites to yield beneficial conditions for the reverse transformation of Ni-rich austenite phase. From the TEM and APT results as displayed in Figure 3, it can be observed that the co-precipitation microstructure of the reversed austenite phase is accompanied by Ni diffusion and Cu-rich nanoprecipitates [96].

**Figure 4:**
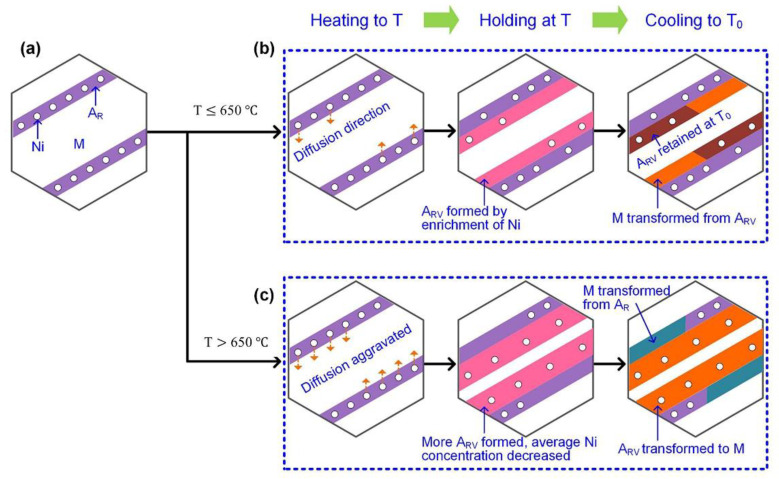
Schematic illustrations for reverted austenite formation treated by diverse heating temperature [4]: (**a**) The as-welded specimen, (**b**) applying heat treatment temperature below 650 °C, (**c**) applying heat treatment temperature above 650 °C. (Reprinted with permission from Ref. [4]. Copyright 2019 Elsevier).

Chao Zhang et al. [97] have demonstrated that the Ni favors migrating into the Ni_3_Ti precipitates and martensitic matrix, and the Ni_3_Ti precipitates impede the transformation of reverted austenite and considerably decrease the toughness under aging treatment at 300 to 500 °C. The inhibition effect of Ni_3_Ti precipitates is weakened with the increment of aging temperature. Moreover, harnessing the composition fluctuation and nano-segregation from the supersaturated solution to obtain the ultra-high strength steel was achieved [98]. The mechanism of microstructure transformation including the fraction and stability of austenite for post-heat treatment in martensitic stainless steel was illustrated, which is mainly controlled by the diffusion behavior of the Ni element as shown in Figure 4 [4].

## 3. Microstructure and Properties after Typical Heat Treatment Processing

### 3.1. Nanoprecipitates in PHSS

**Figure 5:**
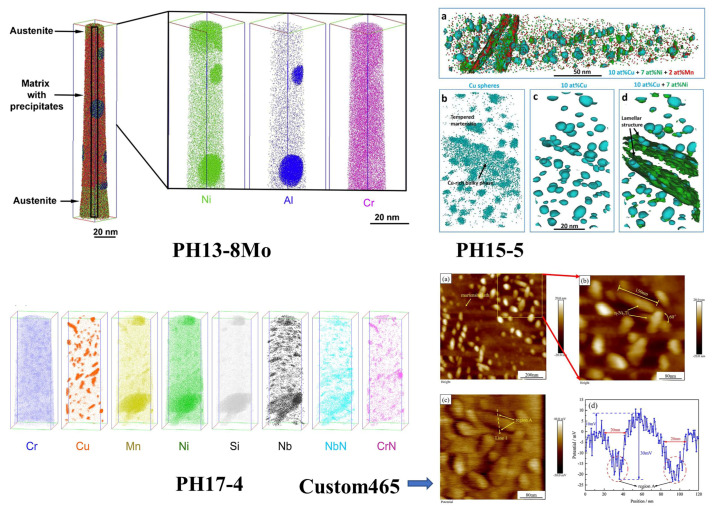
Characteristic nanoprecipitate microstructures in various PHSS: PH13-8Mo [15]; (Reprinted with permission from Ref. [15]. Copyright 2010 Elsevier); (**a**–**d**) PH15-5 [107] (Adapted from Ref. [107].); PH17-4 [11] (Adapted from Ref. [11].); ((**a**)–(**d**)) Custom 465 [64]. (Reprinted with permission from Ref. [64]. Copyright 2019 Elsevier).

Advanced PHSS not only has a combination of ultra-high strength and acceptable toughness, but also the steel has certain anti-corrosion properties [99,100]. Additionally, the steels with excellent properties require the adjustment of the microstructure, especially in the formation of nano-precipitates. It is well known that dislocations can pass through precipitates by cutting or bypassing the mechanism, and the mechanical properties are affected by the interaction between precipitates and dislocations, which is closely related to the size of precipitates. Furthermore, various types of strengthening phases in the existing representative PHSS have been investigated [101,102,103], such as B2-NiAl particles in PH13-8Mo steel [104,105], the Cu-rich cluster in PH17-4 and PH15-5 steels [83,106,107,108], and Ni_3_Ti precipitates in Custom 465 steel [64], etc. [109,110]. As shown in Figure 5, it provides an encyclopedic understanding solution to PHSS.

PH13-8Mo stainless steel is strengthened by the B2 type NiAl intermetallic phase. Through the three-dimensional reconstruction of the chemical composition atomic region, it can be observed that NiAl precipitates after aging at 575 °C for 100 h, and NiAl precipitates and reverts to an austenite form on the matrix at the same time [15,111]. APT inspection found that Cu-rich precipitation and other nano precipitation (such as rich niobium and NbN/CrN precipitation) were fully formed in PH17-4 steel after heat treatment at 590 °C for 20 min, and the distribution of Cu-rich precipitation on the alloy was more uniform than at 480 °C [11]. Meanwhile, the quantity density and size of Cu-rich particles and Cr-rich precipitates were found to increase with the increasing service time, indicating the hardening effect of PH17-4 steel under the nuclear power plant environment [112]. The Cu precipitation promotes the formation of reverted austenite via the diffusion of elements [113]. The preferential corrosion nucleation regions with the weak passive film are prone to form at the interface between the Ni_3_Ti precipitates and the matrix in Custom 465 steel [64]. The nanoprecipitates including B2-NiAl phase, R phase (Fe_3_Mo_2_), and austenite phase in Custom 475 steel were systematically characterized and analyzed utilizing high-resolution transmission electron microscopy [41]. Meanwhile, the maximum hardening effect was realized via aging treatment at 520 °C for 4 h. Primary NiAl precipitates cause a slow and gradual increase in the hardening effect. Among these strengthening precipitation intermetallic phases, the strengthening effect induced by Ni_3_Ti is the most apparent due to the addition of Ti alloying element, and the strengthening effect of Cu-rich precipitation is relatively weak [114]. Furthermore, PHSS hardened by different co-existing precipitates demonstrated that the desired properties can be integrally optimized. Multiple precipitates of Cu precipitates, Ni_3_Ti precipitates, and MC carbides were observed in novel ultra-high strength stainless steel with fully martensitic microstructures via tuning the composition and heat treatment processing [115]. The Cu-rich precipitates, Mo-rich precipitates, and Ni_3_(Ti, Al) phase are detected after heat treatment in maraging steels [64,116].

### 3.2. Effect of Heat Treatment on the Microstructure and Properties in PHSS

Individual heat treatment processes can significantly modify the microstructure features in PHSS, such as the length and width of hierarchical lath martensite, the fraction of austenite (residual austenite and reversed austenite), and the size and distribution of precipitated phases, which will have a considerable impact on the strength and toughness of PHSS. At the same time, the heat treatment process will determine the segregation and enrichment of alloying elements in steel, which will also alter the performance of PHSS to a certain extent. It is crucial to interpret the relationship between the specific heat treatment process and the internal microstructure and precipitated phase of PHSS. The microstructure and properties of PHSS under different heat treatment conditions were investigated from a large number of literatures. The heat treatment routes of several typical PHSS are summarized in Table 3.

The heat treatment of PHSS usually embodies the solution treatment (ST) followed by the aging treatment (AT). Optimizing the heat treatment schedules has been demonstrated as a powerful strategy for enhancing tailored properties. A very high or very low ST temperature will induce the change in microstructure. In general, the solution treatment temperature (STT) should be selected as 900–1100 °C, with a dwell time of 1–2 h, and then cooled to below $M_{s}$ temperature. If necessary, the cryogenic treatment should be carried out to obtain complete martensite. At the same time, the PHSS is subjected to the aging treatment temperature (ATT) at 480–620 °C [117,118]. The aging process promotes the precipitation of fine and dispersed nanoprecipitates; namely, the intermetallic strengthened phase is regulated to obtain the highest strength and satisfactory comprehensive mechanical properties. The parameters of heat treatment are usually determined according to the targeted comprehensive mechanical properties.

**Figure 6:**
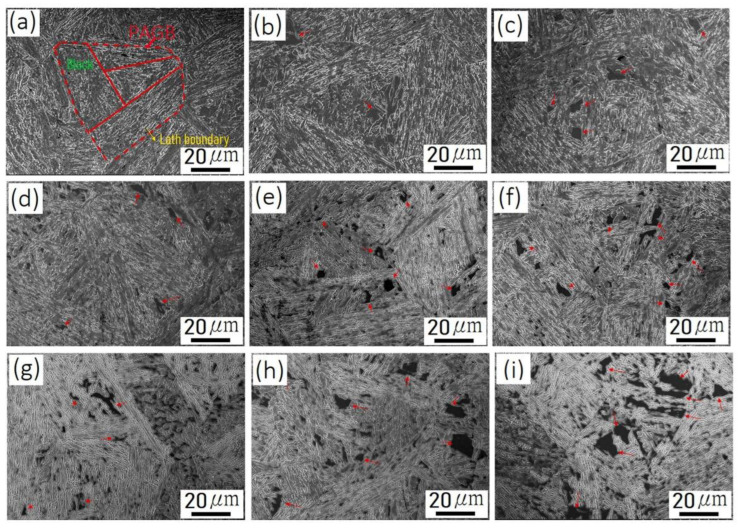
Hierarchical microstructures in Custom 465 steel aged at (**a**–**c**) 480 °C, (**d**–**f**) 560 °C, (**g**–**i**) 640 °C for (**a**,**d**,**g**) 1 h, (**b**,**e**,**h**) 4 h, and (**c**,**f**,**i**) 8 h, respectively. The reverted austenite within the martensitic matrix is marked by the red arrows [125]. (Reprinted with permission from Ref. [125]. Copyright 2022 Elsevier).

In the previous investigations, the aging heat treatment of Custom 465 steel mostly adopts the temperature of 480–648 °C and the aging time of 4 h [122,123,124]. The evolution of Ni_3_Ti precipitates with a rod-like shape and reverted austenite has been a research hotspot in PHSS. Figure 6 displays the representative hierarchical microstructures in Custom 465 steel aged at different conditions corresponding to 480–640 °C for 1, 4, and 8 h, respectively [125]. The hierarchical microstructural features of martensitic steel consist of prior austenite grain, martensite groups, packets, blocks, and lath with the same variant, which can be identified clearly. Moreover, the dimension of Ni_3_Ti precipitates coarsened with the aging temperature and time imply that the precipitation behavior conformed to the thermal activation mechanism. Additional reverted austenite is obtained aging at 520 °C and the presentence of reverted austenite induced the inhomogeneous distribution of Ni_3_Ti particles and a broader hardness distribution. Ronald Schnitzer et al. [111] have studied the dynamic mechanical properties of PH13-8Mo steel after solution annealing at 900 °C for 1.5 h followed by the aging treatment at 575 °C for different times. The results show that the reverted austenite exhibits the instability of dynamic mechanical behavior, and the transformation of austenite into martensite is detected. Although no precipitates are detected, the hardness of the PHSS is enhanced to a certain extent at the preliminary stage of aging treatment [105,126,127]. At the same time, an improvement in yield strength occurs after 10 h of aging, and the long-term aging results show that the strengthening response is significantly faster at 400 °C. The spinodal decomposition of Fe-rich and Cr-rich phases was detected at the aging time of 5000 h [127]. L.W. Tsay et al. explored the sulfide stress corrosion cracking behavior and mechanisms of PH13-8Mo steel after aging at 482–593 °C. The content of reverted austenite determines the hardness and strength value, which indicates that the specimen after the aging treatment at 593 °C displays better stress corrosion cracking resistance, although the hardness is lower than the other aged samples [128]. In addition, the fracture modes of PHSS change after hydrogen charging, and hydrogen embrittlement is relieved after proper heat treatment techniques [129,130]. The dissolution and transformation of Ni_3_Ti precipitates and the enhanced stability of reverted austenite mainly diminish the hydrogen susceptibility after the over-aging heat treatment at 593 °C [130].

**Figure 7:**
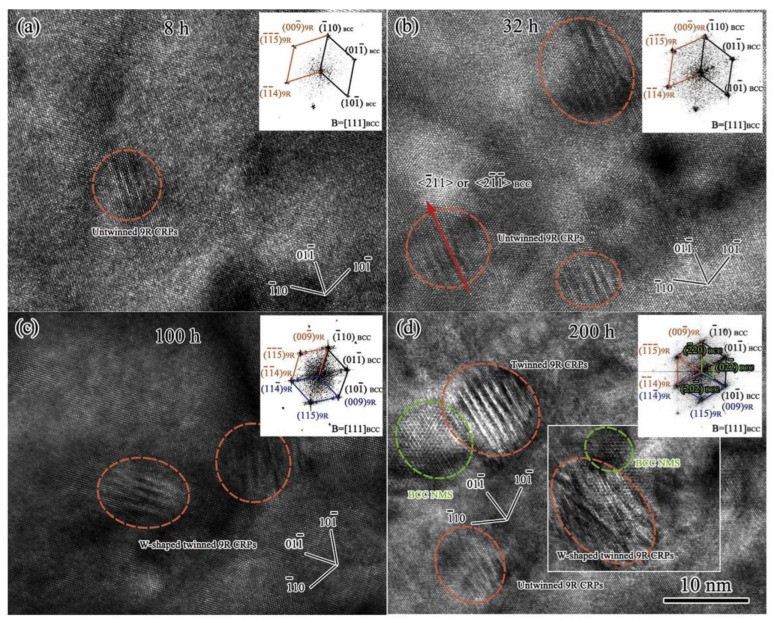
HRTEM micrograph and insert FFT results of precipitates taken along ${\left[111\right]}_{\alpha -\mathrm{Fe}}$ during aging: (**a**) Only one Cu-rich precipitate with un-twinned 9R structure after aging for 8 h; (**b**) three un-twinned 9R Cu-rich precipitates treated for 32 h; (**c**) W-shaped twinned 9R Cu-rich precipitates aging for 100 h; (**d**) precipitates co-precipitated with twinned and W-shaped twinned 9R Cu-rich precipitates for 200 h [88]. (Reprinted with permission from Ref. [88]. Copyright 2018 Elsevier).

**Figure 8:**
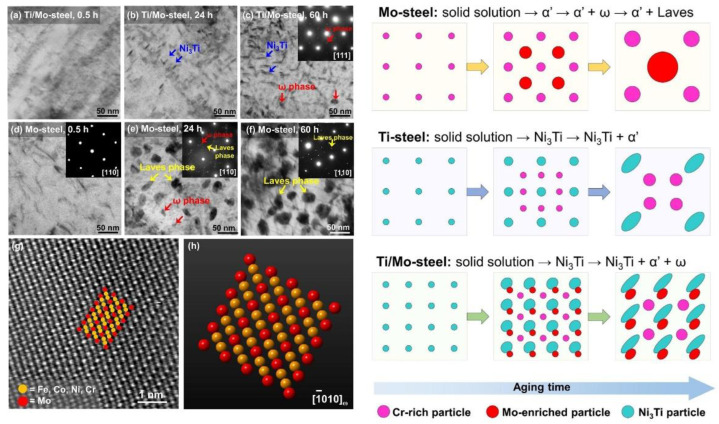
TEM and STEM measurements of the Ti/Mo- and Mo-steels under distinct aged processes and corresponding to the evolution mechanisms: (**a**) Ti/Mo-steel, 0.5 h, (**b**) Ti/Mo-steel, 24 h, (**c**) Ti/Mo-steel, 60 h, (**d**) Mo-steel, 0.5 h, (**e**) Mo-steel, 24 h, and (**f**) Mo-steel, 60 h. (**g**) Presents an HAADF-STEM micrograph of a ω precipitate in (**e**), and (**h**) displays the simulated atomic structure of ω phase [131]. (Reprinted with permission from Ref. [131]. Copyright 2021 Elsevier).

M.C. Niu et al. [131] have investigated the collaborative effects of Mo, Ti, and Cr on the precipitation behavior and mechanical properties of PHSS using experimental and computational approaches. The precipitation sequence of Ni_3_Ti, Mo-rich, and Cr-rich precipitates during aging for 0.5, 2, and 60 h at 500 °C were revealed. In addition, the mean radius and volume fraction of Cr-rich particles in Ti/Mo steel is 1.8 nm and 3.1%, respectively. Zeming Wang et al. [88] studied the evolution of multiple nanoprecipitates and their interactive effect on the mechanical properties of PH17-4 PHSS aged at 450 °C for 0.5–200 h. Figure 7 shows the HRTEM micrograph and insert FFT results of precipitates. The initial clear hardening effect is strengthened by Cu-rich clusters with a core-shell structure. The co-existing of Ni, Mn, Si, and Nb-rich precipitates and Cu-rich clusters with an un-twined 9R structure as the extension of aging time as well as the evolutions of Cr-rich regions were analyzed. The diameters of Cr-rich domains and Cu-rich clusters increase as a function of aging time up to 200 h, relatively, the density number of Cu-rich clusters is decreased, and the strength increment of Cr-rich regions can compensate for the strength loss due to Cu-rich clusters. Generally, the steels are subjected to the solution treatment at 1038–1040 °C of quenching and followed by the aging treatment at different temperatures for different times [132,133]. The as-solutioned samples are mainly composed of lath martensitic microstructures with a small fraction of δ-ferrite. The toughness enhanced (from 15 to 50 ft-lb) and the strength decreased (from 1379 to 999 MPa) are observed with the increase in ATT from 480 to 621 °C [133]. The two commonly applied aging processes are aging conditions at 482 °C for 1 h or aging at 593 °C for 4 h. PH17-4 stainless steel with the highest strength and hardness is obtained under the condition of under-aging treatment, and the over-aging process can guarantee the toughness and ductility of the PH17-4 steel. The TRIP effect of the austenite phase can optimize the ductility of PHSS when the aging temperature exceeds 580 °C [134]. Meanwhile, with the increase in aging time, the strengthening effect of the Cu-rich phase will be weakened due to over-aging. However, the contribution of Cr-rich precipitates can largely compensate for the reduction in the hardening effect of the Cu-rich phase under the aging temperature of 480 °C [11]. When the temperature of solution treatment exceeds 495 °C, the intergranular corrosion sensitivity of the PH17-4 specimens is significantly improved through electrochemical measurements and evaluations [31]. The co-precipitation effect of Ni_3_Ti, Mo-rich, and Cr-rich precipitates promotes the strength of the PHSS with a value of 1.8 GPa after aging at 500 °C for 60 h. The corresponding precipitation and evolution mechanisms of PHSS are presented in Figure 8 [131].

The relevant literature has demonstrated that the evolution behavior in mechanical properties with the aging process is attributed to the balanced effect of precipitates, phase transformation, and austenite morphology features. The study of PH13-8Mo steel results uncover that the NiAl strengthening particles first displayed a steady growth followed by a clear coarsening of about 9 nm at 593 °C for 5 h and the hardness dropped significantly, corresponding to the over-aging state for PH13-8Mo steel [5]. The highest hardness of 39 HRC and the least toughness for Custom 450 steel were observed for 2 h at 565 °C due to the possible precipitation effect. Moreover, the existence of continuous reversed acicular-austenite promotes the higher toughness aged for 4 h, and additional globular austenite and thickened acicular austenite decrease the toughness [35]. The transformation products of B2-NiAl precipitates, R phase, and austenite phase in Custom 475 steel are observed, and these precipitates contribute different strengthening effects relying on the aging temperature. The formation of B2-NiAl precipitates (2–5 nm) acted as primary strengthening precipitates inducing a slow incremental hardening effect at 480 °C. Effective hardening was gained by aging at 520 °C and the peak hardness (601 HV) was obtained when the steel was aged for 4 h along with the co-existence microstructure of fine B2 particles and medium-sized R phase (20–30 nm) [41]. The Ti-containing steel displays an elemental substitution in the type of precipitates during aging, accelerating the growth of particles and a significant reduction in hardness [61]. It is demonstrated that the Cu-rich precipitates and dislocation density are two factors regulating the evolution of the yield strength of the tempered martensite of the PH15-5 steel. The lath of martensite coarsening occurs during the aging treatment. However, the high-angle boundaries are more important for the strength of the martensite [110]. The as-aged martensitic microstructure has little influence on hardness in the PH17-4 steel, and the age-hardening behavior in PH17-4 steel is similar to the typical PHSS alloys [118].

The precipitation and evolution of reversed austenite have been investigated after various aging heat treatment processes. During tensile deformation, metastable reversed austenite transforms into martensite, which greatly improves plasticity and toughness. The Cu-assisted steel containing 12.4% reversed austenite displays a good combination of strength (yield strength of 1330 MPa), ductility (15%), and impact toughness (58 J) [96]. The volume fraction of reverted austenite is about 1–2% in Corrax PHSS alloy, which is consistent with a predicted value of 2.5% [119]. The peak strength at 580 °C was obtained, corresponding to the aging time of 0.25 h. The impact toughness showed a lower value of 151 J at the peak-aged state and enhanced upon over-aging of the material. The effect of inverted austenite on strength and impact toughness is weaker than the effect of Cu-rich precipitates [12]. The granular austenite and elongated austenite are observed after aging at 575 °C. As the aging time increases, the growth of reversed austenite leads to the dissolution of the adjacent NiAl precipitates [15]. From tensile results, it can be estimated that about 40% of the reduction in strength (from 1249 to 1000 MPa) during aging can be originated from the existence of reversed austenite. With an increasing fraction of reverted austenite, an increased strain-hardening exponent was analyzed, and reverted austenite is not mechanically stable during dynamical tensile measurements [111].

**Figure 9:**
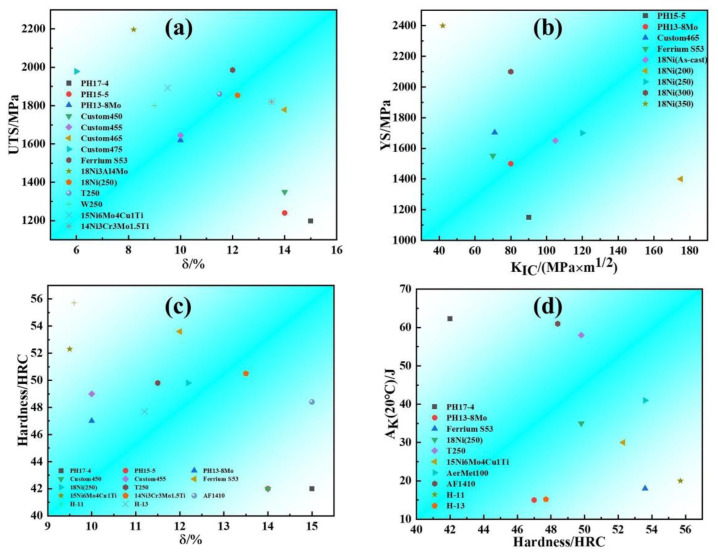
Mechanical properties of typical precipitation hardened stainless steel [135,136,137,138]: (**a**) Ultimate tensile strength-elongation; (**b**) yield strength-fracture toughness; (**c**) hardness-elongation; (**d**) impact work-hardness.

Furthermore, we summarize the mechanical properties of common PHSS and several high-strength steels. The mechanical properties of typical PHSS at ambient temperature, such as the tensile properties, hardness, impact work, and fracture toughness, are plotted in Figure 9 [135,136,137,138]. A novel ultra-strong maraging steel strengthened by Ni(Al,Fe) precipitates was developed based on a minimal lattice misfit strategy, achieving a strength of 2.2 GPa and uniform elongation with the value of 3.8% upon aging for 3 h at 500 °C [135]. Ultra-high strength/hardness and moderate toughness balance of mechanical properties for PHSS are highly desired. The results display that the existing PHSS has sufficient strength and ductility, but there is still room for improving the toughness. Compared with the traditional martensitic stainless steel, future work is required to further improve the toughness without sacrificing strength and plasticity, which is more challenging. Meanwhile, we also present the electrochemical corrosion behavior parameters of PHSS after different heat treatments in various corrosive mediums, and the characteristic indicators including corrosion potential ($E_{corr}$), corrosion current density ($I_{corr}$), pitting potential ($E_{pit}$), passivation current density ($I_{pass}$), and corrosion rate are tabulated in Table 4. The results show that great endeavors of corrosion behavior evaluation for PHSS are focused on the Cl-containing medium, especially in the 3.5 wt% NaCl solution. The data of the corrosion current density show that the PH17-4 stainless steel with a lower value possesses better electrochemical corrosion response than other PHSS as displayed in Table 5.

## 4. Effects of Different Preparation Methods on Microstructure and Properties

**Figure 10:**
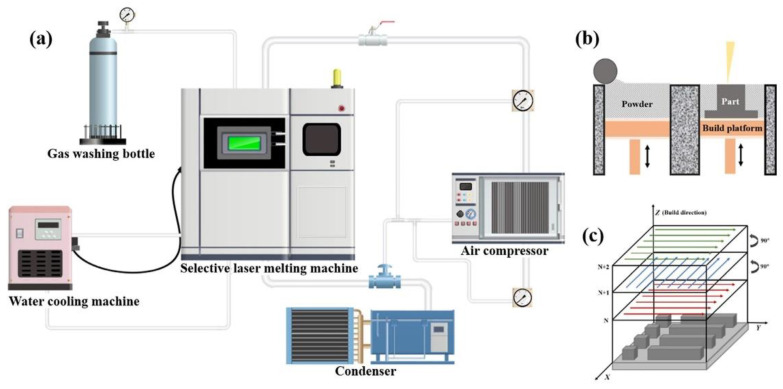
The basic flowchart of SLM: (**a**) Parts of the experimental apparatus, including selective laser melting machine, water-cooled machine, condenser, air compressor, and gas washing bottle; (**b**) working sketch of SLM; (**c**) print strategy of specimens [157]. (Reprinted with permission from Ref. [157]. Copyright 2022 Elsevier).

Different fabricating approaches will also have a non-negligible impact on mechanical and corrosion behavior, which is not limited to the adjusting alloy composition and heat treatment parameters. At present, many state-of-the-art manufacturing methods have been reported in the literature, mainly focusing on AM technology [145,146,147]. Generally, the methods of fabricating PHSS include casting and wrought procedures [11,122] and powder metallurgy [148,149]. Conventional cast PHSS is processed and deformed by forging to improve the microstructure, supplemented by the appropriate heat treatment to obtain the desired properties. Jan Kazior et al. [147] have examined the properties of PH17-4 steel fabricated by the powder metallurgy method, and they found that adding temperature is a very sensitive parameter to obtain high strength with satisfactory ductility. Additive manufacturing (AM) approaches as disruptive technology [8,32,150,151,152,153,154], due to their near-net-shape feature, cost-effective, and customized flexible design for complicated parts, are widely introduced to prepare PHSS gradually across multi-industries. The processing methods of AM for PHSS are still in their infancy. SLM uses computer-aided design as a digital information source and combines fine metallic powders with laser beams to fabricate three-dimensional metal parts. The SLM method of AM approach supports the manufacture of dense components with superior mechanical properties when the parameters are optimal. Meanwhile, the ultra-low carbon content facilitates crack-free addictive manufacturing of the PHSS [8,155]. Among the AM techniques, laser powder bed fusion (L-PBF) technology with near-net-shaping dimensions has become a conventional preparation method for geometrically complex structural parts due to its flexible geometric design and high spatial accuracy [155,156]. On the one hand, the PHSS with fine grain microstructure, which originated from the higher cooling rates, is obtained based on the L-PBF processing. On the other hand, it is arduous to produce parts with tailored structures or properties employing typical alloys [142,157]. Defects such as porosity, the loss of alloying elements, and cracking were observed, which hinder the process of future industrial application. Meanwhile, the heterogeneous microstructures and residual stress were retained during the cyclic heating and cooling process in manufacturing. Therefore, it is important to better understand the microstructure characteristics of PHSS prepared by the L-PBF method and its impact on properties. The target samples with a special dimension were fabricated utilizing commercial SLM apparatus (AFS-M120). The apparatus was equipped with a 500 W fiber laser with a focal laser beam diameter of 0.075 mm and a wavelength of 1070 nm. The argon environment was adopted to shield the processing and diminish contamination, as schematically shown in Figure 10a. The printing path always maintained 90 degrees angle rotation between the following layers Figure 10c [157]. A broad range of print parameters was selected to study the print capability of the PHSS.

**Figure 11:**
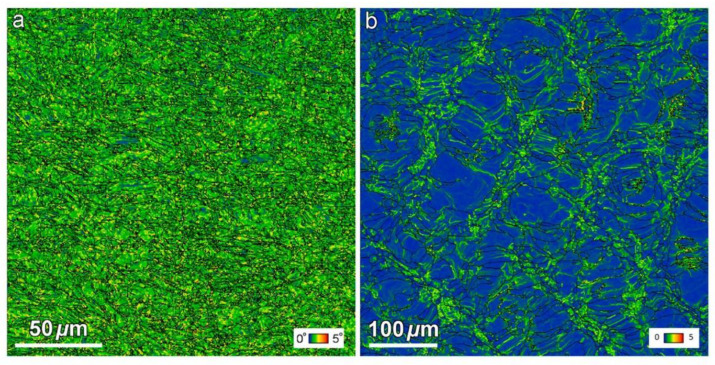
KAM results of the as-built PH17-4 PHSS with (**a**) martensitic microstructure and (**b**) ferritic microstructure [158]. (Adapted from Ref. [158]).

The optimization of melting parameters including scanning speed and energy density are of great importance to obtaining valuable parts. S. Sabooni et al. [158] have explored the influence of post-heat treatment on the microstructure evolution and mechanical properties in L-PBF of PH17-4 steel from two different feedstock powders. The results displayed that the full martensite phase and ferritic microstructure were observed in Figure 11, which mainly depends on the chemical composition of powders. The martensitic microstructural samples displayed accelerated age-hardening behavior compared with the samples with ferritic, which can be elucidated by the improved diffusivity of precipitation elements caused by the increment of grain boundaries and the lath martensitic microstructures with higher dislocation density. The martensitic microstructure of PH17-4 steel is easy to undergo the reversion of austenite under direct aging treatment. The reverted austenite enhances the ductility of heat-treated samples. The Kernel Average Misorientation (KAM) images illustrate the higher stored energy in the martensitic microstructures, which provides the driving force for the formation of the precipitated phase.

**Figure 12:**
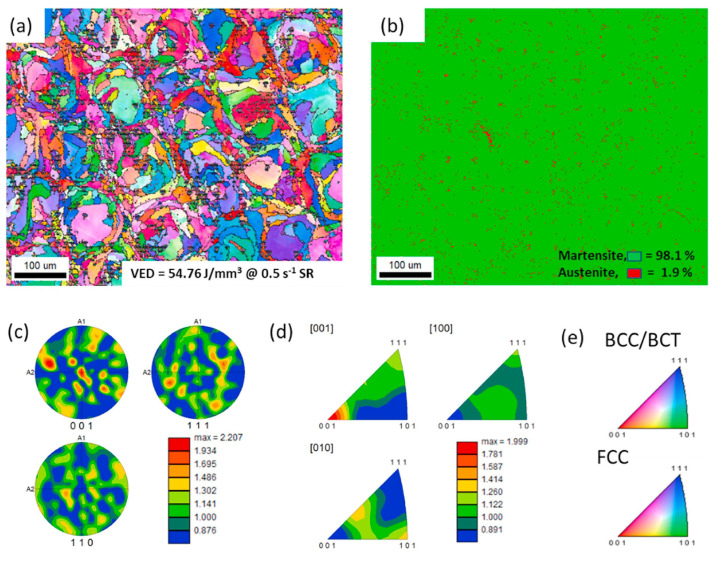
EBSD micrographs of nanoindentation region (at 0.5 s^−1^ strain rate) of as-printed L-PBF PH17-4 PHSS part with VED = 54.76 J/mm^3^ [159]: (**a**) IPF image in the (X–Y) plane perpendicular to the print direction, (**b**) phase map, (**c**) texture pole figure, (**d**) inverse texture pole figure, and (**e**) IPF color maps of phases. (Reprinted with permission from Ref. [159]. Copyright 2022 Elsevier).

**Figure 13:**
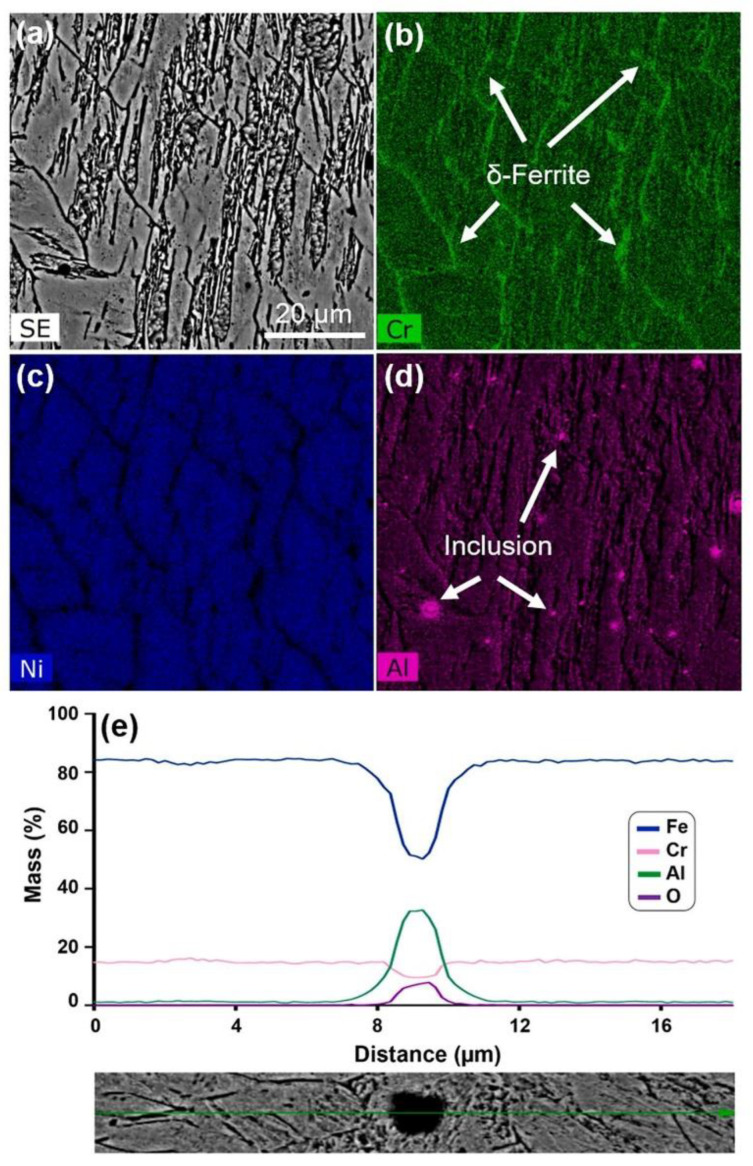
The chemical elemental mapping and line profiles selected from the as-printed microstructure of wire arc addictive manufactured PH13-8Mo steel [160]: (**a**) SEM image; (**b**–**d**) chemical elemental mappings; (**e**) the line profiles of selected region. (Reprinted with permission from Ref. [160]. Copyright 2022 Elsevier).

Mohammad Jashim Uddin et al. [159] considered the process parameters including volumetric energy density (VED), scanning speed, and hatch distance in L-PBF approaches. The fraction of the austenite phase is only 1.9% after low VED in the as-printed PH17-4 part according to the EBSD quantitative analysis. The substantial alterations of micro-mechanical properties, yield, and maximum shear strength associated with the strain rates were detected in the proton-irradiated L-PBF PH17-4 parts as displayed in Figure 12. It can be noticed that grains become slightly finer as a result of radiation in irradiated specimens with the same VED. Mahya Ghaffari et al. [160] studied the microstructure and mechanical properties of PH13-8Mo steel manufactured by wire arc additive manufacture (WAAM) approaches. The typical microstructures are composed of the vermicular and lathy remnant δ-ferrite distributed on the fine martensitic matrix coupled with a low percentage of retained austenite. Meanwhile, the anisotropic mechanical properties are strongly related to the columnar growth of δ-ferritic microstructure with a significant texture during solidification. Furthermore, the spherical Al-rich oxide inclusion particles were characterized, which can be ascribed to the retained oxygen in the protecting environment and possible moisture on the raw material as shown in Figure 13.

**Figure 14:**
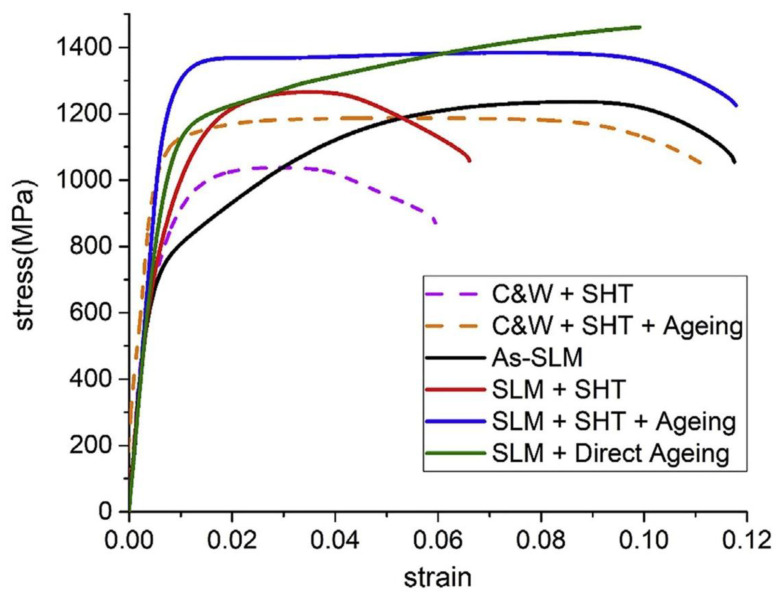
Tensile response of SLM processed samples compared with conventional casting techniques subjected to various heat treatments [162]. (Reprinted with permission from Ref. [162]. Copyright 2019 Elsevier).

Many efforts have been made to investigate the influence of printing strategy and heat treatment schedule on the mechanical and corrosion properties of PHSS due to the anisotropy of the microstructure and properties of the parts after AM methods. Printing orientation has a significant effect on low-cycle fatigue and high-cycle fatigue properties. Post-heat treatment has been proven to be an effective strategy to enhance the tensile behavior and low-cycle fatigue property of PH17-4 steel. However, the influence of the microstructural impurity on the low-cycle fatigue property is not conspicuous, but it is more sensitive to the high-cycle fatigue behavior. The un-melted region is an important factor to consider the deterioration of properties beyond the density [161]. Tzu-Hou Hsu et al. [162] revealed the mechanism of oxide dispersion strengthening of PH17-4 steel by introducing geometrically necessary dislocations. Meanwhile, the post-heat treatment can trigger the martensitic microstructures to the formation of reverted austenite or the Cu-rich precipitates. The results of mechanical properties show that the tensile properties and hardness of SLM are better than the conventional casting and forging techniques, as shown in Figure 14. Tao Zhou et al. regulated the formation of reverted austenite and the precipitation of nanoscale precipitates under post-heat treatment and obtained a distinguished balance between strength and ductility based on the wire arc additive manufacturing method. Nanoscale precipitation hardening promotes the improvement in high strength; however, the reverted austenite with high stability and fine grain size is beneficial for ductility [149]. Chuanfeng Wu et al. [163] unfolded the heterogeneous mechanical properties along the building direction in direct laser-deposited PH17-4 steel, and the acceptable deformation compatibility in the microstructure at the top of specimen was discussed. The cooperative effect of strain partitioning hardening and the austenitic transformation-induced plasticity promotes the superior balance of strength and ductility [149].

**Figure 15:**
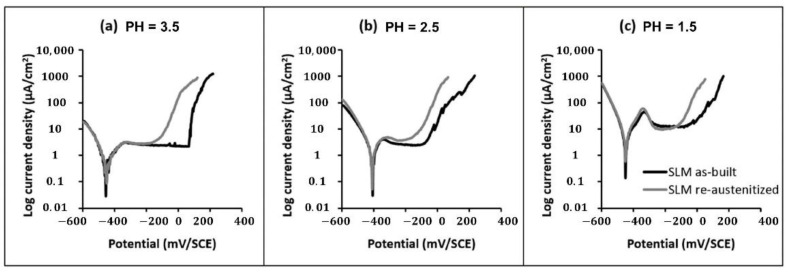
Comparison of the electrochemical behavior of the as-built SLM-ed steel and the re-austenitized SLM-ed steel in NaCl solution at different acid atmospheres: (**a**) pH = 3.5, (**b**) pH = 2.5, and (**c**) pH = 1.5 [150]. (Reprinted with permission from Ref. [150]. Copyright 2020 Elsevier).

It was revealed that the microstructure of as-built PH17-4 via the SLM method corresponds to the full ferrite rather than the martensitic phase. However, the general corrosion behavior of the sample after heat treatment exhibits distinguished properties from the wrought martensitic steel (Figure 15) [150], and the precipitation of Cu-rich particles was observed in the steel prepared by SLM methods and conventional techniques [32]. PH17-4 steel manufactured by SLM consists of 72% metastable austenite and 28% martensite phase, and the distinguished mechanical properties result from the strain-induced transformation of austenite and microstructural features of dual phase [164,165]. The difference of corrosion behavior with microstructural inhomogeneity was systematically studied [32,113]. The size and morphology of the microstructure are observed to vary from the top and side perspectives of building directions employing the direct-metal-laser-sintering (DMLS) method [166]. The results illustrate that the H900 samples display the highest anti-corrosion properties than the other heat treatment conditions, and the enhanced corrosion and mechanical properties can be achieved by optimizing the heat treatment process on the fabricated PH15-5 steel. An insignificant growth of lath size, a reduction in dislocation density, low angle grain boundary, and low residual stress level on the side view were demonstrated to contribute to the improvement in the corrosion response of the side surface [32].

## 5. Applications of Multi-Scale Computational Simulations and ML in Modeling the Relationships among Composition, Microstructures, Process, and Properties of PHSS

**Figure 16:**
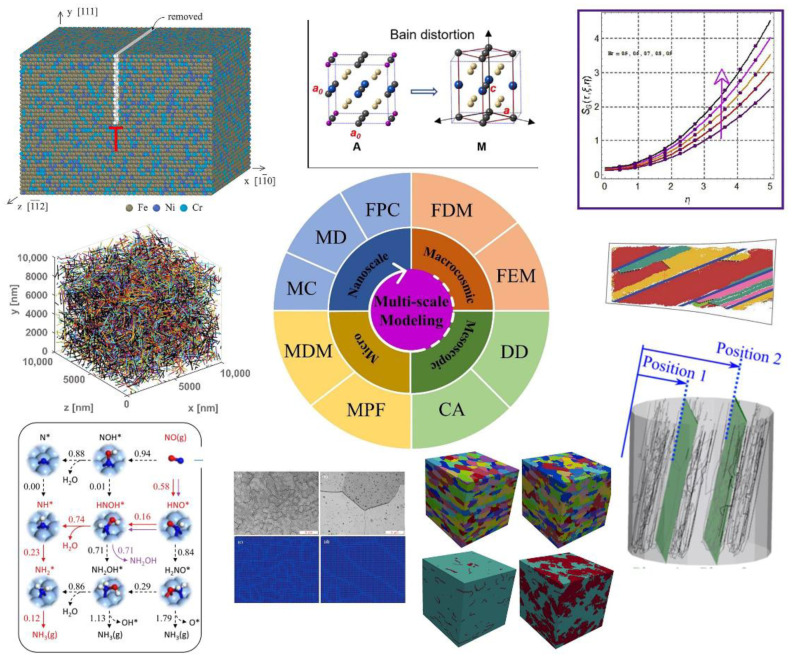
Various modeling and simulation methods in materials science across multi-scales [167,168,169,170,171,172,173,174,175,176]. Abbreviation in the figure: Finite difference method (FDM), finite element modeling (FEM), dislocation dynamic (DD), cellular automaton (CA), microscopic phase field (MPF), microscopic dynamic modeling (MDM), Monte Carlo (MC), molecular dynamics (MD), first principles calculation (FPC). (Reprinted with permission from Ref. [167]. Copyright 2022 Elsevier; Reprinted with permission from Ref. [168]. Copyright 2019 Elsevier; Reprinted with permission from Ref. [169]. Copyright 2021 Elsevier; Reprinted with permission from Ref. [171]. Copyright 2021 Elsevier; Reprinted with permission from Ref. [172]. Copyright 2022 Elsevier; Reprinted with permission from Ref. [173]. Copyright 2021 American Chemical Society; Reprinted with permission from Ref. [174]. Copyright 2023 Elsevier; Reprinted with permission from Ref. [175]. Copyright 2020 Elsevier; Reprinted with permission from Ref. [176]. Copyright 2022 Elsevier).

Generally, the traditional material design method based on trial and error is very cumbersome; therefore, it is urgent to carry out the modeling optimization. In recent years, the multi-scale calculation and simulation methods of materials have been developed rapidly and widely harnessed in the material design and mechanism research of new materials, which provides an efficient way for the development of new PHSS. Figure 16 displays diverse computational and simulation approaches used in materials science from the macroscale, microscale to the nanoscale, corresponding to the typical simulation approaches and examples in materials science investigations, such as the finite difference method (designated as FDM), finite element modeling (FEM), dislocation dynamic (DD), cellular automaton (CA), phase field especially in microscopic phase field (MPF), microscopic dynamic modeling (MDM), Monte Carlo (MC), molecular dynamics (MD), and first principles calculation (FPC).

The obtained FDM is determined through a numerical strategy. Physical characteristics of fluid flow, temperature, entropy optimization, and concentration have been illustrated. Variations of parameters are graphically investigated [167]. The microstructural evolution and deformation behavior of alloy were studied in detail based on the crystal plasticity-based FEM [168]. Meanwhile, the deformation simulations included the prediction of stress-strain for PHSS employed by FEM approaches [152,169]. The inherent localization physical mechanisms of mechanical behaviors manifested by DD simulation are well illustrated from the analysis of the results, which can shed a further understanding at the dislocation level [169]. A CA algorithm was utilized to simulate the nucleation and grain growth of microstructural evolutions of PH17-4 steel during investment casting [170]. The framework of ML combined with CA was presented with remarkable accuracy to investigate the static recrystallization microstructural evolution of FCC polycrystalline materials, accelerating the innovation of novel/enhanced materials [171]. The alteration of atomic microstructure morphology maintains the consistency between the experimental and MPF simulation results. Meanwhile, the MDM and MD approaches at the microscale were adopted to reveal the dynamic reaction mechanisms [172,173]. Moreover, the FPC, MD, and some MC simulations were conducted to uncover the inherent behavior and mechanism from the atomic point-of-view and further control the tailored properties of developed materials [47,174,175,176,177,178]. Arpana S. Murthy et al. [47] have illustrated the segregation of Co atoms from the Cu-rich particles for PH17-4 steel utilizing the FPC method via the energy-minimum criterion. Tian et al. [177] have investigated that the addition of Co increases Fe-Fe ferromagnetic interactions and promotes the formation of chromium-rich clusters in Fe. Therefore, combined with the characteristics of PHSS, the targeted properties of PHSS can be efficiently predicted in the PHSS design with the assistance of multi-scale calculation and simulation techniques.

**Figure 17:**
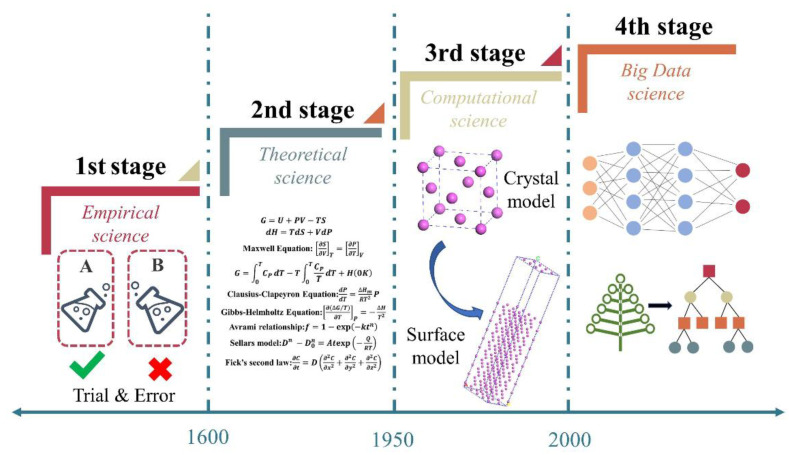
Development in the materials science divided into four stages: Empirical, theoretical, computational, and big-data.

**Figure 18:**
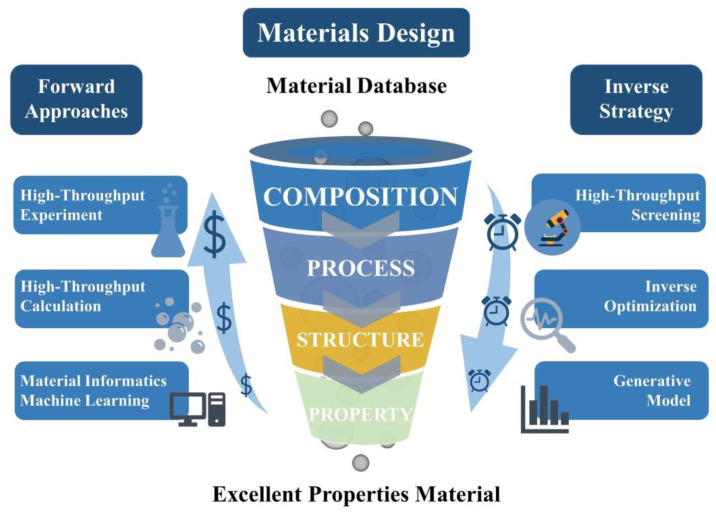
Facing ML to enhance material innovation and development based on bidirectional strategies.

As shown in Figure 17, the development of materials science has experienced a research paradigm based on pure experience, followed by the development of theoretical science represented by mathematical description, thermodynamics, and material dynamics equations. Since 1950, computational science, phase field dynamics, and other methods that have gradually developed into computational materials science methods have been used to study materials and explain the evolution mechanism of materials’ microstructure by solving differential equations. Computational techniques should be used to carefully study the PHSS multi-dimensional composite space. Consequently, material informatics are applied to extract knowledge from existing large data sets and establish a model to lay a foundation for materials design with the advent of the big-data era. As a material information technology, data driven science can be realized through data mining, ML, and mathematical optimization. It can use existing databases and high-throughput data based on forward and reverse methods, as shown in Figure 18, to discover the relationship among composition, process, microstructure, and performance, forming a new method to understand materials and facilitate material design. In addition, different relevant algorithms are adopted to establish models to achieve high accuracy in material research. The most credible strategy to combine ML algorithms with the prediction of mechanical properties is to design physically presentative descriptors and take advantage of the existing database of materials’ properties as training data, developing reliable and affordable materials with high performance.

There are many types of alloying elements, and the additional content of alloying elements as well as the interaction between various elements would increase the complexity of the experiment. The traditional trial and error approaches are simple in facing the dilemma of low efficiency. C.E. Campbell et al. [178] established an empirical Equation (1) about $M_{S}$ via considering the chemical and mechanical energy changes with different alloying elements, which provides theoretical guidance for the alloy design of PHSS:

(1) $$Ms\left(K\right)=818-33000\times C_{c}+200\times C_{Al}+700\times C_{Co}-1400\times C_{Cr}-1300\times C_{Cu}-2300\times C_{Mn}-500\times C_{Mo}\;-400\times C_{Nb}-1300\times C_{Ni}-700\times C_{Si}+300\times C_{Ti}+400\times C_{V}$$

With the vigorous development of computational science, it is a new trend to examine the influence of alloying elements on the properties of materials by leveraging algorithms to model rationally. Additionally, common ML algorithms were adopted to investigate the intrinsic relations of materials especially in steels, including random forest (RF), linear regression (LR), support vector regression (SVR), multi-layer perceptron (MLP), convolutional neural network (CNN), and K-nearest neighbor (KNN) [179,180,181,182,183,184,185,186]. Yupeng Diao et al. [179] proposed a ML prediction model for comprehensive properties and successfully employed the efficient global optimization algorithm to optimize multi-objective mechanical properties for carbon steels. The corrosion rate of low-alloy steel in marine environments was effectively predicted via feature selection and feature descriptor creation [183].

**Figure 19:**
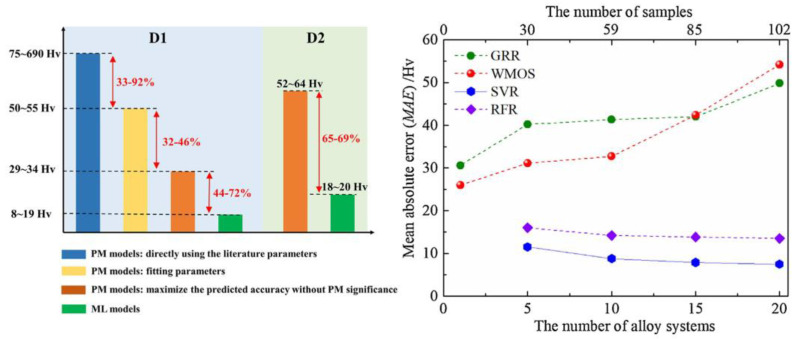
Semi-quantitative and quantitative analysis of the prediction accuracy for ML and PM methods [186]. (Reprinted with permission from Ref. [186]. Copyright 2021 Elsevier).

Physical metallurgical (PM) method has been employed as an efficient strategy to develop distinguished mechanical properties and illustrate the mechanisms of strength increment. Furthermore, Chunguang Shen et al. [186] introduced PM parameters into ML modeling and established a ML model guided by PM, and these physical parameters can be easily obtained, which are assisted by thermodynamic software calculations. The precision of best prediction results of the PM model is apparently lower than the ML model. It can be seen that with the expansion of the data set, the overfitting result gradually weakens, as shown in Figure 19. Finally, the authors successfully established the ML model with high prediction accuracy and strong generalization ability through regression modeling and genetic algorithm optimization, and verified the accuracy of the model in predicting ultra-high PHSS through experiments. In addition, a genetic optimization framework was constructed to the cultivation of stainless steel strengthened by Ni_3_Ti nanoprecipitates coupled with thermodynamic calculations and PM theories [187]. Meanwhile, microstructural features extraction, composed of the size and morphology factors of defects in the L-PBF fabricated PH17-4 stainless steel, was carried out and the correlations between defect characteristics and fatigue properties were accurately obtained based on the SVR framework [188]. Therefore, an accessible pathway consisting of ML and multi-scale simulation method is validated to exploit high-performance PHSS. Moreover, the feed-back ANN algorithm was adopted for modeling the available flow curves of PH17-4 steel and the aging hardening parameters for PH17-4 steel were optimized by ANN and genetic algorithm, demonstrating the capability of ML [27,189]. 

## 6. Summary and Outlook

In the present work, we focus on the establishment of the composition-microstructure-properties model of PHSS to urgently develop novel precipitation hardening stainless steels with superior mechanical properties. We over-reviewed the development history of representative PHSS. The influence of the composition fluctuation of general alloying elements on the microstructure and properties is discussed, and the research progress of the microstructure and properties of PHSS after the heat treatment process is summarized and illustrated, especially in the aspects of mechanical properties to guide the optimization of the heat treatment process. The relationships between precipitation strengthening nanoparticles and the microstructure and properties of steel were revealed, including typical B2-NiAl precipitates, Ni_3_Ti phase, Cu-rich clusters, and other strengthening phases. A novel material with multiple co-existing particles was indicated to achieve comprehensive performance. 

Compared to traditional fabrication techniques, the advancement of AM in terms of machine capabilities and process parameters has resulted in the development of parts. The emergence of AM techniques provides strong support for the manufacturing and application of PHSS parts, especially for improving the microstructure and performance of PHSS steel prepared by SLM. At present, the AM technology of alloys is still in the immature stage. The parallel development of AM technology based on the traditional method of fabricating alloys provides convenience for the application of PHSS. There is still an enormous space for SLM to fabricate steel parts due to the drawbacks of PHSS, such as anisotropy, macro and micro defects, and residual stress after the existing processes.

The development of new techniques and new approaches ensures the future exploration of PHSS with application potential. Moreover, it is necessary to employ ML strategies in materials science to extract the data from the results of experimental physical metallurgy and multi-scale simulation approaches, rather than trial-and-error methods, to comprehensively and efficiently design tailored PHSS with excellent mechanical properties. Additionally, future investigations will focus on developing reliable and robust databases and modeling the assessable correlations of chemical composition, hot work processes, microstructure, and properties. Machine learning and multi-scale simulation methods are used to reveal the relationship among the fraction and features of precipitates, processes, and properties to achieve accurate regulation of the precipitates. Meanwhile, attention should be paid to the precision heat treatment parameter control that takes into account fluctuations in composition by combining intelligent algorithms.

Therefore, ML coupled with multi-scale simulation approaches and experimental methods undoubtedly exhibits a high-efficiency direction toward the development of novel PHSS with high performance, since ML has a strong capability to solve the intrinsic quantitative relationship between the microstructure of composition/process properties in the PHSS system. The prosperity of ML applications in PHSS design is poised to provide the perspective for a novel paradigm in integrated multi-scale computational materials science as a whole.

## Figures and Tables

**Table 1:** The main features of typical precipitation hardened stainless steels with developed year [34,35,36,37,38,39,40,41,42,43,44,45,46].

Type	Main Features	Designed Year	Reference
PH17-4	Simple heat treatment process; Excellent corrosion performance; Easy to weld; Limited cold working capacity; Low impact toughness.	1948	Reddy, V.V., et al., 2015.
Custom450	Good combination of strength, toughness, and anti-corrosion properties; Cu-rich precipitate is the main precipitated phase.	1961	Bhavsar, V., et al., 2022.
PH15-5	The Ni and Cr contents are modified based on PH17-4 steel; Lath martensitic microstructures; The strength and toughness are enhanced.	1965	Fu, P., et al., 2019.
PH13-8Mo	Ultra-high strength and hardness; Excellent stress anti-corrosion properties.	1968	W.M. Garrison et al., 1991.
PH17-7	Semi-austenitic PHSS; Surgical instruments, pressure vessels, and aerospace parts.	1977	Ziewiec, Aneta et al., 2016.
1RK91	Strengths exceeding 3000 MPa; Retaining superior ductility.	1991	NILSSON, J.O., et al., 1994.
Custom 465	Anti-corrosion properties have been further improved; Costs are greatly rising due to the high content of alloying element.	1997	V. Prasath et al., 1991.
Custom 475	Better tensile strength, fracture toughness, and manufacturability; Ti-free, more Mo and Co than Custom 465 steel.	2003	Huang C., et al., 2021.
Corrax steel	Highly corrosive injection mold steel; Simple heat treatment process; Prepare medical devices.	-	Asgari H., et al., 2018; Güldibi, Ahmet Serdar et al., 2020.
Ferrium S53	Reduce the content of Cr and Ni; Increase the content of C and Co; Strengthening effect through the M_2_C type nano-coherent precipitates; Excellent strength and anti-corrosion properties.	2008	Yangpeng Zhang et al., 2018; Pioszak, G.L. et al., 2017; Seo, JY., et al., 2017.

**Table 2:** Chemical composition in representative precipitation hardening stainless steels (wt%) [35,39,41,44,45,46,59,60,61,62,63,64,65].

Types	Ni	Cr	Mo	Si	Mn	Others	Fe	Reference
PH17-4	4.39	16.89	-	0.41	0.79	0.42 Nb 3.12 Cu	Bal.	Kochmański, P., et al., 2006.
Custom 450	6.58	14.79	0.77	0.23	0.78	0.41 Nb 1.54 Cu	Bal.	Bhavsar, V., et al., 2022.
PH15-5	4.6	15.1	-	0.6	0.69	0.25 Nb 3.3 Cu	Bal.	I. Zukerman et al., 2007.
PH13-8Mo	8.54	12.76	0.78	0.58	0.39	3.39 Al	Bal.	Schober, M., et al., 2009.
PH17-7	7	17	-	1	1	-	Bal.	Xu, X.L., et al., 2008.
1RK91	9	12	4	0.15	-	0.9 Ti 0.3 Al 2.0 Cu	Bal.	NILSSON, J.O., et al., 1994.
Custom 465	11.07	12.1	1.07	0.1	0.02	1.83 Ti	Bal.	Bonora, R.G., et al., 2014; Li Wang, et al., 2019.
Custom 475	8	11	5	0.5	0.5	8.5 Co	Bal.	Huang C., et al., 2021.
Corrax steel	9.2	12	1.4	0.4	0.4	1.6 Al	Bal.	Hadadzadeh, A., et al., 2019.
Ferrium S53	5.5	10.0	2.5	-	-	14.0 Co 1.0 W 0.3 V	Bal.	Yangpeng Zhang et al., 2018; Pi-oszak, G.L. et al., 2017; Seo, JY., et al., 2017.

**Table 3:** Classical heat treatment regime of PHSS [35,36,38,41,43,44,62,63,100,117,118,119,120,121].

Types	STT (°C)	STt (h)	ATT (°C)	ATt (h)	Reference
PH17-4	1040	1	450	4	Wang Z., et al., 2017.
	1040	0.05	480	1	Hsiao C.N., et al., 2002.
	1040	0.05	620	1	
PH15-5	1170	1	500	2	Tao Zhou et al., 2008.
	1038	-	480	-	Fu, P., et al., 2019.
PH13-8Mo	850	0.5	525	3	Xu, X.L., et al., 2008.
	940	2	550	4	Snir Y, et al., 2018.
	940	2	600	4	
PH17-7	1050	3	580	2.5	Xu, X.L., et al., 2008.
	1050	3	640	2.5	
	760	1	510	1	Ziewiec, Aneta et al., 2016.
Custom 450	1040	1	565	2	Bhavsar, V., et al., 2022.
	1040	1	565	4	
Custom 465	1050	0.5	538	3	Bonora, R.G., et al., 2014.
	1050	0.5	593	3	
Custom 475	1100	1	480	8	Huang C., et al., 2021.
	1100	1	520	4	
Corrax steel	850	0.5	525	3	S. Höring et al., 2009.
	850	0.5	525	12	
	850	0.5	400	4	Güldibi, Ahmet Serdar et al., 2020.
	850	0.5	600	4	
1RK91	1100	-	475	4	Stiller, K. et al., 1998.
Ferrium S53	1080	1	680	8	Yangpeng Zhang et al., 2018.
	1085	1	482	12	Yangpeng Zhang et al., 2019.

**Table 4:** Corrosion properties of PHSS after various heat treatment processes [113,139,140,141,142,143,144].

Alloy	ST	AT	$E_{corr}$ (V)	$I_{corr}$ (µA/cm^2^)	$E_{pit}$ (V)	$I_{pass}$ (µA/cm^2^)	Corrosion Rate (mm/yr)	Corrosive Medium	Reference
PH17-4	1038 °C × 1 h	480 °C × 1 h			0.173	0.27		3.5% NaCl	Shoushitari et al., 2010.
	1038 °C × 1 h	550 °C × 4 h			0.205	0.18			
	1038 °C × 1 h	620 °C × 4 h			0.124	0.1			
PH17-7	1750 F × 10 min + (−100) F × 8 h	950 F × 1 h	−0.396					3.5% NaCl	Repukaiti, Reyixiati 2017.
PH15-5	1900 F × 1 h	900 F × 1 h	−0.422						
PH15-5	1040 °C × 0.5 h	1025 F × 4 h	−0.275	0.126		0.398		3.5% NaCl	Qiang Guo 2015.
Direct Metal Laser Sintering-PH15-5	1040 °C × 0.5 h		−0.295	21.64				3.5% NaCl	Avula I., et al., 2021.
	1040 °C × 0.5 h	900 F × 4 h	0.114	0.035					
	1040 °C × 0.5 h	925 F × 4 h	−0.279	3.692					
16Cr-5Ni-1Mo	1050 °C × 1 h	400 °C × 4 h	0.130				1.156	6% FeCl_3_	R. Abdel-Karim 2004.
		475 °C × 4 h	0.108				1.504		
		550 °C × 4 h	0.077				1.102		
		625 °C × 4 h	0.072				1.120		
		700 °C × 4 h	0.080				1.165		
		750 °C × 4 h	0.091				1.263		
	1050 °C × 1 h	625 °C × 1 h	0.071				1.035		
		625 °C × 6 h	0.089				1.103		
		625 °C × 8 h	0.085				1.120		
		625 °C × 16 h	0.07				1.312		
13 Cr	1020 °C×0.5 h		−0.46	0.24	−0.051	1.54		0.1 M NaCl	Bonagani, S.K., et al., 2018.
		300 °C × 2.5 h	−0.557	0.38	−0.08	3.59			
		550 °C × 2.5 h	−0.585	7.22	−0.585				
		700 °C × 2.5 h	−0.591	8.59	−0.152	93.76			
PH15-5	1038 °C × 0.5 h			0.403	0.06			3.56% NaCl	Sagar Sarkar et al., 2020.
	1038 °C × 0.5 h	482 °C × 1 h		0.235	0.015				
	1038 °C × 0.5 h	621 °C × 4 h		0.307	0.16				

**Table 5:** Corrosion current density of various PHSS [28].

Steels	PH17-4	PH15-5	PH13-8Mo	1RK91	Custom 465	Custom 475	Ferrium S53
*I_corr_* (µA/cm^2^)	1.409	2.176	4.522	4.301	13.326	17.087	20.271

## Data Availability

The date presented in this study are available on request from the corresponding author.

## Abbreviations

Abbreviation	Definition
PHSS	Precipitation hardening steel
ML	Machine learning
MD	Molecular dynamics
PHS	Precipitation hardening steel
MP	Microstructure and properties
SLM	Selective laser melting
TEM	Transmission electronic microscopy
APT	Atom probe tomography
SAED	Selected area electron diffraction
EDS	Energy dispersive spectroscopy
EBSD	Electron Backscattered Diffraction
ST	Solution treatment
AT	Aging treatment
STT	Solution treatment temperature
STt	Solution treatment time
ATT	Aging treatment temperature
ATt	Aging treatment time
HRTEM	High Resolution Transmission Electron Microscope
FFT	Fast Fourier Transformation
TRIP	Transformation-induced plasticity
STEM	Scanning transmission electron microscope
HAADF	High-angle annular dark-field
AM	Addictive manufacture
L-PBF	Laser powder bed fusion
KAM	Kernel Average Misorientation
VED	Volumetric energy density
WAAM	Wire arc additive manufacture
DMLS	Direct-metal-laser-sintering
FDM	Finite difference method
FEM	Finite element modeling
DD	Dislocation dynamic
CA	Cellular automaton
MPF	Microscopic phase field
MDM	Microscopic dynamic modeling
MC	Monte Carlo
FPC	First principles calculation
FCC	Face-centered cubic
B2	Ordered body centered cubic structure
RF	Random forest
LR	Linear regression
SVR	Support vector regression
MLP	Multi-layer perceptron
CNN	Convolutional neural network
KNN	K-Nearest Neighbor
PM	Physical metallurgy
$M_{S}$	Martensite starts temperature
YS	Yield strength
UTS	Ultimate tensile strength
δ	Elongation
$K_{IC}$	Fracture toughness
$A_{K}$	Charpy impact energy (work)
$E_{corr}$	Corrosion potential
$I_{corr}$	Corrosion current density
$E_{pit}$	Pitting potential
$I_{pass}$	Passivation current density
